# Supplementary material for: Deep learning-based prediction of chemical accumulation in a pathogenic mycobacterium
Source: bioRxiv. 2024 Dec 16:2024.12.15.628588. Preprint. [Version 1] doi: 10.1101/2024.12.15.628588 (PMC11702553; doi:10.1101/2024.12.15.628588)
Supplement: Supplement 1 [file NIHPP2024.12.15.628588v1-supplement-1.pdf]

bioRxiv preprint doi: https://doi.org/10.1101/2024.12.15.628588; this version posted December 16, 2024. The copyright holder for this preprint (which was not certified by peer review) is the author/funder, who has granted bioRxiv a license to display the preprint in perpetuity. It is made available under aCC-BY 4.0 International license.

Supplemental Figures

bioRxiv preprint doi: https://doi.org/10.1101/2024.12.15.628588; this version posted December 16, 2024. The copyright holder for this preprint (which was not certified by peer review) is the author/funder, who has granted bioRxiv a license to display the preprint in perpetuity. It is made available under aCC-BY 4.0 International license.

A

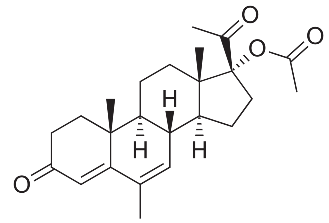

megestrol acetate: 6.92

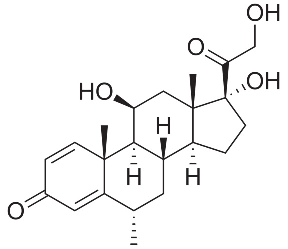

methylprednisolone: 3.95

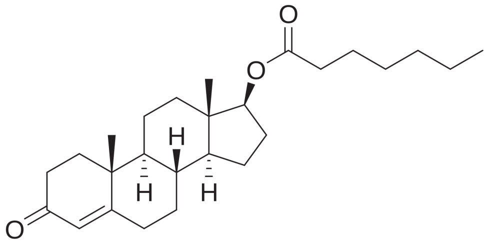

testosterone enanthate: 0.78

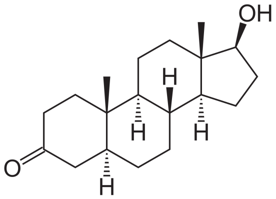

dihydrotestosterone: 1.91

Supplemental Figure 1

bioRxiv preprint doi: https://doi.org/10.1101/2024.12.15.628588; this version posted December 16, 2024. The copyright holder for this preprint (which was not certified by peer review) is the author/funder, who has granted bioRxiv a license to display the preprint in perpetuity. It is made available under aCC-BY 4.0 International license.

Supplemental Figure 1. Steroid-like compounds display a wide range of accumulation. (A) Chemical structures and $\log_{10}$ relative accumulation of selected steroid-like compounds in *M. abscessus*.

bioRxiv preprint doi: https://doi.org/10.1101/2024.12.15.628588; this version posted December 16, 2024. The copyright holder for this preprint (which was not certified by peer review) is the author/funder, who has granted bioRxiv a license to display the preprint in perpetuity. It is made available under aCC-BY 4.0 International license.

A

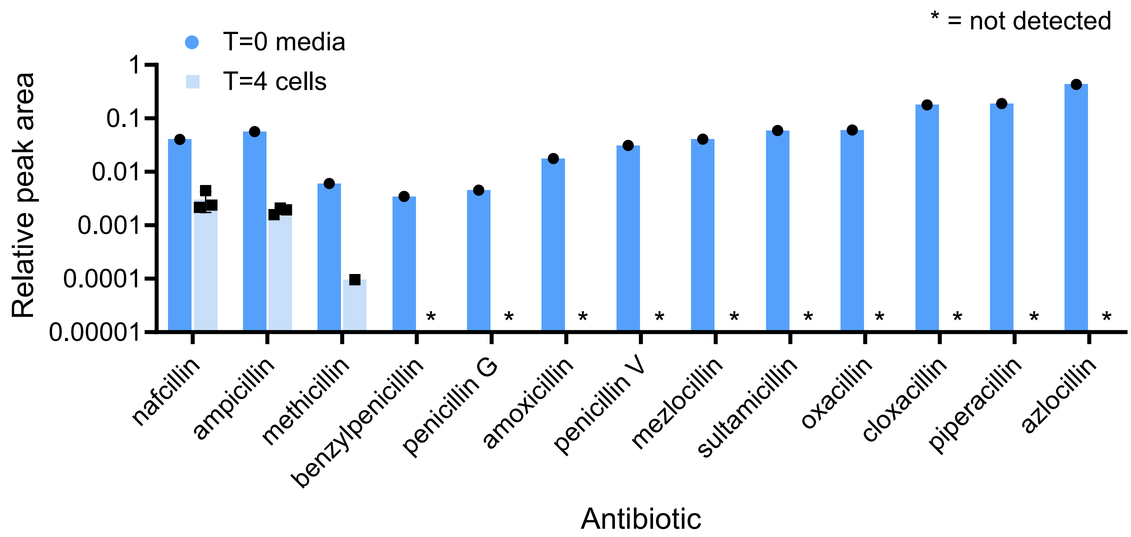

B

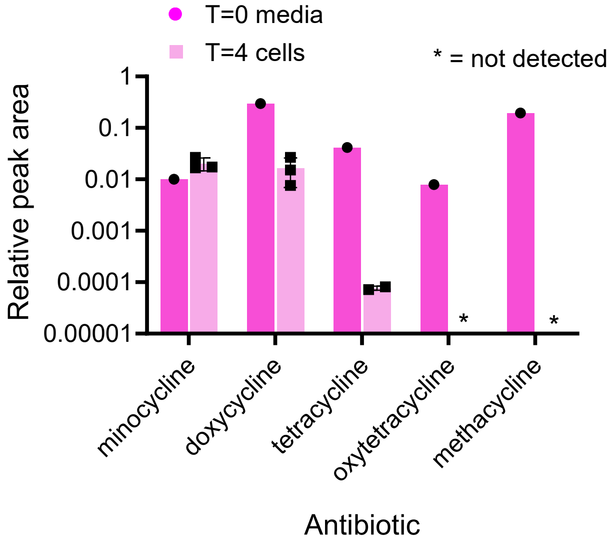

C

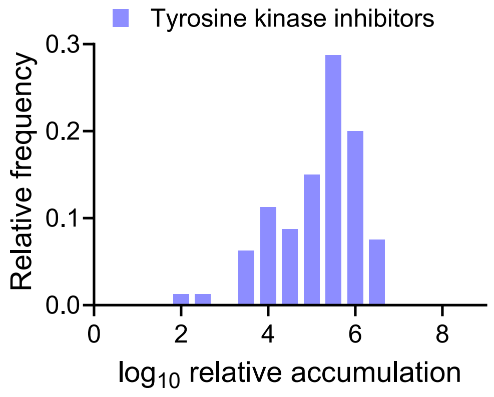

D

	In top 10%	Not in top 10%
Tyrosine kinase inhibitors	24	56
Non-tyrosine kinase inhibitors	134	1368

p<0.0001

Supplemental Figure 2

bioRxiv preprint doi: https://doi.org/10.1101/2024.12.15.628588; this version posted December 16, 2024. The copyright holder for this preprint (which was not certified by peer review) is the author/funder, who has granted bioRxiv a license to display the preprint in perpetuity. It is made available under aCC-BY 4.0 International license.

Supplemental Figure 2. Antibiotics known to be degraded by M. abscessus accumulate poorly. (A) Relative peak area in initial media and in cell-associated fraction after 4 hr incubation for the indicated penicillin-related antibiotics. Data represent mean +/- SD. n=3 independent cultures for cell-associated fractions. **(B)** Relative peak area in initial media and in cell-associated fraction after 4 hr incubation for the indicated tetracycline-related antibiotics. Data represent mean +/- SD. n=3 independent cultures for cell-associated fractions. **(C)** Histogram displaying the relative accumulation of tyrosine kinase inhibitors in *M. abscessus*. **(D)** Contingency table categorizing relative accumulation of tyrosine kinase inhibitors versus non-tyrosine kinase inhibitors in M. abscessus. p-value derived from two-sided chi-squared test.

bioRxiv preprint doi: https://doi.org/10.1101/2024.12.15.628588; this version posted December 16, 2024. The copyright holder for this preprint (which was not certified by peer review) is the author/funder, who has granted bioRxiv a license to display the preprint in perpetuity. It is made available under aCC-BY 4.0 International license.

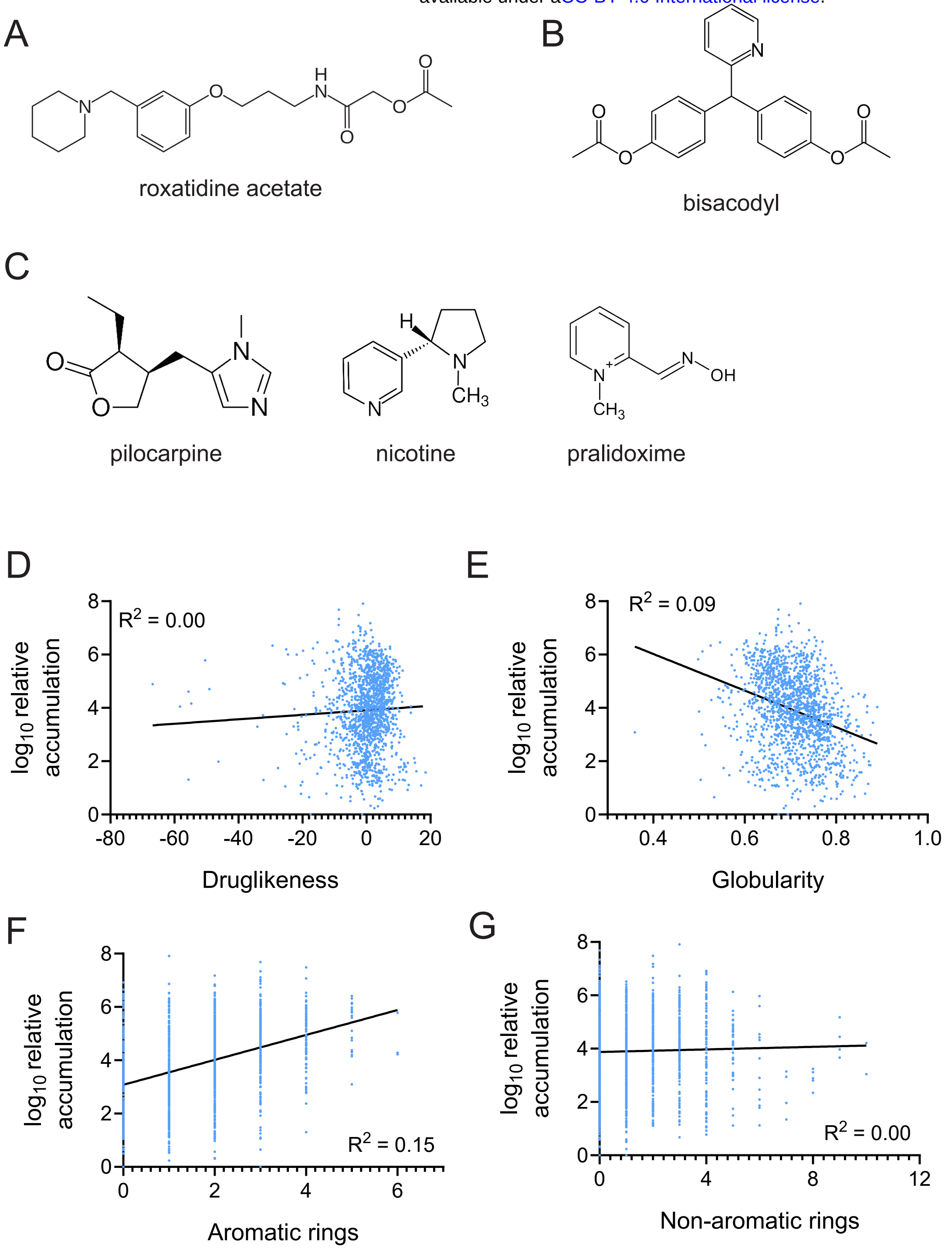

Supplemental Figure 3

bioRxiv preprint doi: https://doi.org/10.1101/2024.12.15.628588; this version posted December 16, 2024. The copyright holder for this preprint (which was not certified by peer review) is the author/funder, who has granted bioRxiv a license to display the preprint in perpetuity. It is made available under aCC-BY 4.0 International license.

Supplemental Figure 3. Physical properties poorly predict chemical accumulation in *M. abscessus*. (A) Chemical structure of roxatidine acetate. **(B)** Chemical structure of bisacodyl. **(C)** Chemical structures of pilocarpine, nicotine, and pralidoxime. **(D)** Correlation between druglikeness **(D)**, globularity **(E)**, aromatic ring content **(F)**, and non-aromatic ring content **(G)** with $\log_{10}$ relative accumulation in *M. abscessus*. R^2 represents the coefficient of determination.

bioRxiv preprint doi: https://doi.org/10.1101/2024.12.15.628588; this version posted December 16, 2024. The copyright holder for this preprint (which was not certified by peer review) is the author/funder, who has granted bioRxiv a license to display the preprint in perpetuity. It is made available under aCC-BY 4.0 International license.

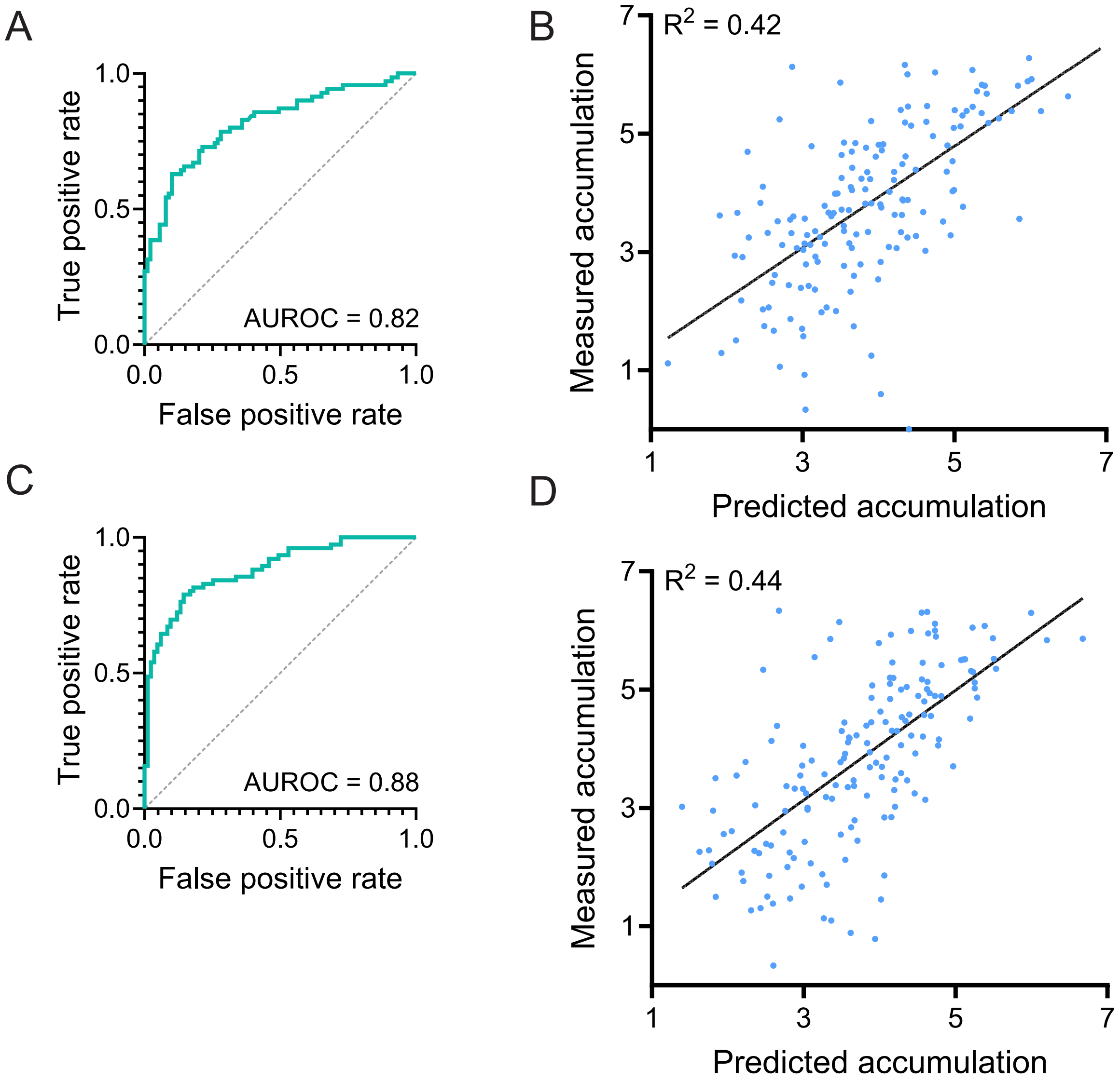

Supplemental Figure 4

bioRxiv preprint doi: https://doi.org/10.1101/2024.12.15.628588; this version posted December 16, 2024. The copyright holder for this preprint (which was not certified by peer review) is the author/funder, who has granted bioRxiv a license to display the preprint in perpetuity. It is made available under aCC-BY 4.0 International license.

Supplemental Figure 4. (A) Receiver operating characteristic (ROC) curve for a classifier that considers a top 50% relative accumulation value to correspond to accumulation. Classifier was generated using different splitting of training data compared to classifier in Figure 4B. **(B)** Correlation of predicted and measured log_{10} relative accumulation of test set compounds by an augmented regression model. Regression model was generated using different splitting of training data compared to regression model in Figure 4E. R^2 represents the coefficient of determination. **(C)** Receiver operating characteristic (ROC) curve for a SMILES-augmented classifier that considers a top 50% relative accumulation value to correspond to accumulation. **(D)** Correlation of predicted and measured log_{10} relative accumulation of test set compounds by a non-augmented regression model. R^2 represents the coefficient of determination.

bioRxiv preprint doi: https://doi.org/10.1101/2024.12.15.628588; this version posted December 16, 2024. The copyright holder for this preprint (which was not certified by peer review) is the author/funder, who has granted bioRxiv a license to display the preprint in perpetuity. It is made available under aCC-BY 4.0 International license.

A

Antibiotic	Antibiotic concentration that prevents 90% of proliferation
Rufloxacin	>128 µM
Danofloxacin	>128 µM
Tosufloxacin	>128 µM
Garenoxacin	>128 µM
Gemifloxacin	>128 µM
Trovafloxacin	>128 µM
Clinafloxacin	>128 µM
Oxolinic acid	>128 µM
Moxifloxacin (control)	16 µM

B

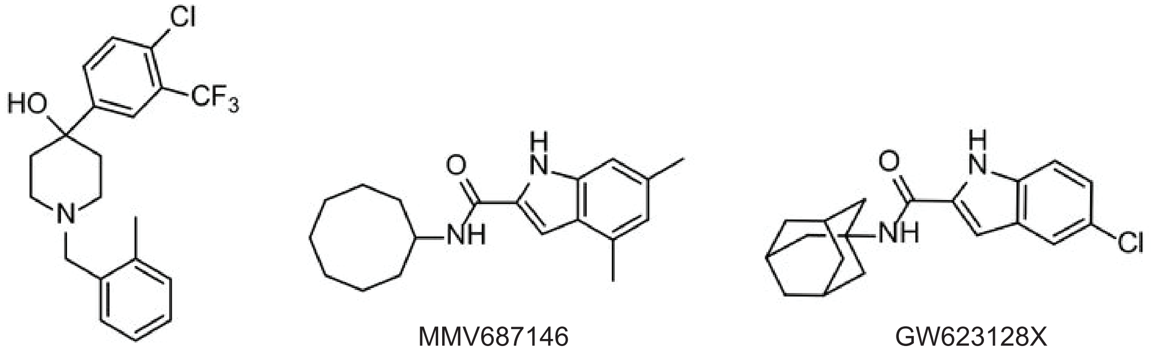

GSK1985270A

MMV687146

GW623128X

C

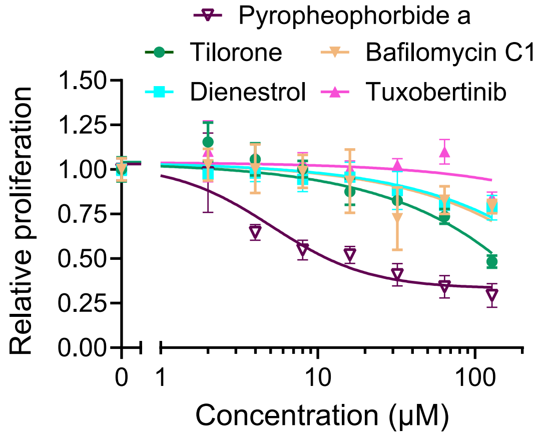

D

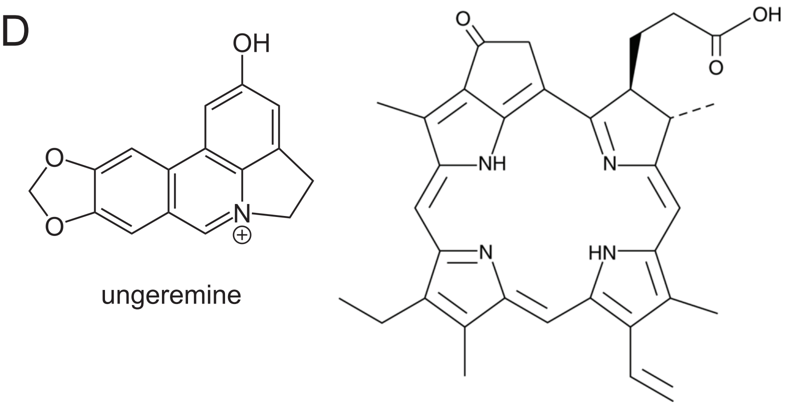

ungeremine

pyropheophorbide a

Supplemental Figure 5

bioRxiv preprint doi: https://doi.org/10.1101/2024.12.15.628588; this version posted December 16, 2024. The copyright holder for this preprint (which was not certified by peer review) is the author/funder, who has granted bioRxiv a license to display the preprint in perpetuity. It is made available under aCC-BY 4.0 International license.

Supplemental Figure 5. Measurement of antibacterial activity of quinolones and high uptake compounds. (A) Antibiotic concentration that inhibits >90% of proliferation of *M. abscessus* as measured by bacterial autoluminescence for 8 quinolone antibiotics measured in Figure 5B as well as clinically used compound moxifloxacin. **(B)** Chemical structures of MmpL3 inhibitors analyzed in Figure 5E. **(C)** Relative proliferation of *M. abscessus* as measured by autoluminescence in the presence of the indicated concentrations of pyropheophorbide a, tilorone, bafilomycin C1, dienestrol, or tuxobertinib. Proliferation is normalized to vehicle-treated condition. Data represent mean +/- SD. n=6 independent cultures for each drug dose. **(D)** Chemical structures of ungeremine and pyropheophorbide a.
